# Enhancement of polyethylene glycol‐cell fusion efficiency by novel application of transient pressure using a jet injector

**DOI:** 10.1002/2211-5463.13557

**Published:** 2023-01-27

**Authors:** Chin Yang Chang, Jiayu A. Tai, Yuko Sakaguchi, Tomoyuki Nishikawa, Yayoi Hirayama, Kunihiko Yamashita

**Affiliations:** ^1^ Department of Device Application for Molecular Therapeutics, Graduate School of Medicine Osaka University Japan; ^2^ Medical Device Division, Industry Business Unit, Safety Strategic Business Unit Daicel Co. Osaka Japan; ^3^ Medical Device Development, Medical Device Division, Industry Business Unit, Safety Strategic Business Unit Daicel Co. Osaka Japan

**Keywords:** antibody engineering, cell fusion, jet injector, PEG, polyethylene glycol, vaccine

## Abstract

Cell–cell fusion involves the fusion of somatic cells into a single hybrid cell. It is not only a physiological process but also an important cell engineering technology which can be applied to various fields, such as regenerative medicine, antibody engineering, genetic engineering, and cancer therapy. There are three major methods of cell fusion: electrical cell fusion, polyethylene glycol (PEG) cell fusion, and virus‐mediated cell fusion. Although PEG cell fusion is the most economical approach and does not require expensive instrumentation, it has a poor fusion rate and induces a high rate of cell cytotoxicity. To improve the fusion rate of the PEG method, we combined it with the pyro‐drive jet injector (PJI). PJI provides instant pressure instead of cell agitation to increase the probability of cell‐to‐cell contact and shorten the distance between cells in the process of cell fusion. Here, we report that this improved fusion method not only decreased cell cytotoxicity during the fusion process, but also increased fusion rate compared with the conventional PEG method. Furthermore, we tested the functionality of cells fused using the PJI‐PEG method and found them to be comparable to those fused using the conventional PEG method in terms of their application for dendritic cell (DC)‐tumor cell fusion vaccine production; in addition, the PJI‐PEG method demonstrated excellent performance in hybridoma cell preparation. Taken together, our data indicate that this method improves cell fusion efficiency as compared to the PEG method and thus has the potential for use in various applications that require cell fusion technology.

## Graphical abstract

Polyethylene glycol (PEG) is commonly used to fuse cells for various applications. In this study, we found that the use of instantaneous pressure can replace the repetitive cell shaking and dilution process in the conventional PEG fusion method and improve overall fusion efficiency and survival rate of fused cells.

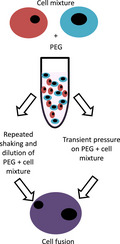

**Abbreviations**
DC vaccines: DC–tumor cell fusion vaccines
DC: dendritic cell
PEG: polyethylene glycol
PEG‐F: PEG‐mediated cell fusion
PJI: pyro‐drive jet injector
PJI‐F: PJI‐mediated fusion

Cell–cell fusion is a biological method to fuse two or more cell types to become a single hybrid cell by either spontaneous or artificial means [1]. It is a useful method with potentially many applications in biotechnology and medical research, such as cancer immunotherapy, antibody engineering, and regenerative medicine in diabetes therapy [2, 3]. In cancer immunotherapy, cell fusion technology is used to fuse dendritic cells (DC) with tumor cells to produce DC vaccines with reactivity against tumor antigens for cancer therapy [4, 5]. Another common cell fusion application is in antibody engineering. Naturally occurring antibody‐producing B cells have finite life span and limited antibody production. In monoclonal antibody research, B cells producing specific monoclonal antibodies are fused with myeloma cell line to produce a hybrid cell line (or hybridoma) that can proliferate and permanently produce and secrete these specific monoclonal antibodies for use in research or large‐scale production. Cell fusion can also be used to produce hybrid hybridomas that produce bispecific antibodies, which target two antigens or epitopes. Therefore, cell fusion is an important tool in biomedical research.

There are several methods to induce cell fusion, the three most commonly used methods include (a) biological method (virus‐mediated fusion), (b) physical method (electrofusion), and (c) chemical‐based methods (PEG fusion) [6]. Each method has its advantages and disadvantages. In biological virus‐based fusion, viruses with fusion glycoproteins on the viral envelope such as Sendai virus or vesicular stomatitis virus are used to initiate fusion between adjacent cells by utilizing the innate viral cell fusion machinery [7, 8]. But cell fusion efficiency using this method is dependent on the initial receptor binding on the target cell surface which brings the viral surface in close contact with the cell surface before the fusion protein can initiate cell fusion [9], so if target cells do not express these binding receptors cell fusion efficiency may be low. Additionally, this method may not be appropriate for fusion involving immune cells due to possible immune stimulation from the virus itself [10]. Electrofusion is a physical method that uses short high voltage electrical pulses to destabilize cell membranes of target cells leading to higher membrane permeability, which improves the probability of the merging of adjacent cell membranes to result in fused cells [11]. This method is both relatively efficient and simple, but it requires costly instrumentation.

Chemical‐based cell fusion method using polyethylene glycol (PEG) is low cost, widely available and does not require expensive machinery [12, 13]. There are a wide range of molecular weights of PEG available, of which the most commonly used for cell fusion is between 1000 and 4000 [14, 15]. In addition to the molecular weight, the concentration of PEG is also an important factor for cell fusion. Higher PEG concentrations produce higher fusion efficiencies, but are also associated with higher cytotoxicity and lower cell viabilities [16, 17]. So, even though PEG is affordable and simple to use, one of the most important problems to overcome is its relatively poor fusion rate.

The jet injector is a needleless drug delivery system with a long history of development [18]. Unlike the conventional syringe injection, jet injector commonly uses instantaneous high pressure to eject a liquid substance to penetrate the skin. Depending on the desired injection depth or injection site, there are various physical means to generate the instantaneous energy to propel injection, such as compressed air or gas, mechanical, pyrotechnic propulsion using gunpowder ignition [19, 20, 21]. More recently, due to the urgent development of new vaccines against SARS‐CoV‐2, jet injectors have garnered increased attention, especially for use to deliver DNA‐based vaccines [22, 23]. Jet devices are not restricted to being vehicles for injection purposes. With more development and modifications, jet devices may provide more potential applications in the biomedical field.

In this article, we try to improve the existing PEG fusion method, especially to the shaking process portion of the cell fusion process. We hypothesize that pressurizing cells within a closed volume vessel can replace the shaking process of the standard PEG fusion method. Based on our results, both mechanical pressure and jet device pressure can replace the shaking process to affect cell fusion. This improvement would not only simplify the PEG cell fusion process, but could also improve overall fusion efficiency and cell viability. We also demonstrated in this study that this new fusion method can be successfully applied to produce DC–tumor cell fusion vaccines and hybridomas, showing that this is a reliable new method and has the potential to improve applications that require cell fusion in the medical field.

## Materials and methods

### Animals and cell lines

Female 6‐week‐old BALB/c mice (CLEA Japan Inc., Tokyo, Japan) were used in the study. All mice were maintained in a temperature‐controlled, pathogen‐free room and were handled according to the approved protocols and guidelines of the Animal Committee of Osaka University (Suita, Japan). All animal experiments performed were approved by The Institute of Experimental Animal Sciences, Faculty of Medicine, Osaka University (Approval number: J007418‐004). NS‐1 and 4T1 mammary carcinoma cell lines were maintained in Roswell Park Memorial Institute 1640 (RPMI1640) medium (Nacalai Tesque Inc., Kyoto, Japan), and MC38 colon adenocarcinoma and B16‐F10 melanoma cell lines were maintained in Dulbecco's Modified Eagle Medium (DMEM) (Nacalai Tesque Inc.). Both RPMI1640 and DMEM complete media were supplemented with 10% fetal bovine serum (BioWest, Nuaille, France) and 0.1 mg·mL^−1^ penicillin–streptomycin mixed solution (Nacalai Tesque Inc.). All cell lines and hybrid cells were cultured at 37 °C in a humidified atmosphere of 5% CO_2_. Experimental animal sacrifice by carbon dioxide animal euthanasia.

### Splenocyte and DC preparation

Spleens were harvested from naive C57BL/6N mice, and the splenocytes derived from the spleens were filtered through a 40‐μm mesh sieve and hemolyzed in hemolysis buffer (Immuno‐Biological Laboratories Co., Ltd., Gunma, Japan). Mouse DCs were isolated by flushing out the bone marrow of the tibia and femur with RPMI1640 medium and then filtered through a 40‐μm mesh sieve. After washing, bone marrow cells were cultured in complete RPMI1640 medium containing 10 ng·mL^−1^ of recombinant mouse GM‐CSF (Wako, Osaka, Japan), as described previously [24]. Culture medium were replaced on Days 2 and 4. On Day 6, nonadherent and loosely adherent proliferating cells were harvested and identified as DCs by evaluating CD11c expression using flow cytometry.

### PEG cell fusion method

Fusion of NS‐1 cells and splenocytes was performed using PEG1500 (Roche, Basel, Switzerland) according to the manufacturer's instructions. Briefly, NS‐1 cells and splenocytes were mixed at a ratio of 1 : 3 (total 8 × 10^6^ cells) and gently agitated by flicking tube 10 times to mix cells, followed by the addition of 100 μL of PEG1500. After PEG addition, the cells were further gently agitated (2 revolutions·s^−1^) in a 37 °C water bath for 90 s and then diluted with equal volume of RPMI1640 medium. Dilution with gentle agitation (2 revolutions·s^−1^) was repeated twice more and then finally rested for 10 min in a 37 °C incubator. For mixed only control group, NS‐1 cells and splenocytes were mixed in the ratio mentioned above along with the addition of PEG1500, then immediately washed with 10 mL medium, then centrifuged to remove supernatant and suspended sample in FACS running buffer. For lower PEG concentration group, manufacturer recommended working concentration of PEG1500 was diluted 1 : 3 with PBS to obtain 25% working concentration PEG.

### Ball drop cell fusion method

Similar to PEG method, NS‐1 cells and splenocytes were mixed at a ratio of 1 : 3 (total 8 × 10^6^) by flicking tube 10 times, followed by the addition of PEG1500. Cell–PEG mixture was loaded into the device container (Fig. S1A) and placed into the ball drop device (Fig. S1C). The ball (50 g) will drop from two different height conditions: 30 and 70 mm (duration: 0.6 and 0.8 ms) to affect cell fusion. After ball drop cell fusion, cell fusion mixtures were returned to RPMI1640 medium immediately for recovery in a 37 °C incubator.

### PJI cell fusion method

PJI fusion was performed using the modified Actranza device (Fig. S1A,B; Daicel Corporation, Osaka, Japan). NS‐1 cells and splenocytes were mixed at a ratio of 1 : 3 and then gently agitated by flicking tube 10 times to mix cells, followed by the addition of 100 μL of PEG1500. Cell–PEG mixture was loaded into the device container (Fig. S1A), and the device was fired to initiate cell fusion (duration: 0.5 ms), and the resultant cell mixture was recovered into RPMI1640 medium in a 37 °C incubator. The fusion cell was observed with a BZ‐X710 microscope (Keyence Corporation, Osaka, Japan). In 3D imaging, the single fluorescent light is a nonfused cell (DiO: green fluorescence or DiD: red fluorescence), and the fluorescent overlap appears yellow as a fusion cell.

### Cell fusion efficiency analysis

To analyze cell fusion efficiency using flow cytometry, NS‐1 cells were prestained with DiR (Thermo Fisher Scientific, Waltham, MA, USA) and splenocytes with BV421‐CD45 (BioLegend Inc., San Diego, CA, USA) prior to fusion and the resultant fusion efficiency was analyzed using CytoFLEX flow cytometer (Beckman Coulter Life Sciences, Brea, CA, USA) and CytExpert software (Beckman Coulter Life Sciences) by calculating the percentage of DiR and BV‐421 double‐positive cells. To determine cell viability after fusion, fused cell mixtures were cultured for 24 h and then stained with trypan blue (Nacalai Tesque Inc.) and the percentage of live/dead cells were counted using TC20 automated cell counter (Bio‐Rad, Hercules, CA, USA). For additional analysis of fusion between different types of cells using PJI, 4T1, MC38, and NS‐1 cells were prestained with 20 μL·mL^−1^ of DiI, DiD, and DiR, respectively (all from Thermo Fisher Scientific) for 20 min, while splenocytes were prestained with FITC‐CD45 (1 : 100 dilution; BioLegend Inc.) for 10 min. After cell staining, 4T1/NS‐1 (1 : 3), MC38/splenocyte (1 : 3), and 4T1/splenocyte (1 : 3) fusion pairs were subjected to PEG or PJI fusion method as mentioned previously.

### DC–tumor hybrid cell vaccine preparation and *in vivo* antitumor effect challenge

Bone marrow‐derived DCs were stimulated with LPS (100 μg·mL^−1^) for 24 h as mentioned previously [4]. B16‐F10 melanoma cells were irradiated at 100 Gray to inactivate cell proliferation. DC–tumor cell fusion was performed by mixing DCs and inactivated B16‐F10 melanoma cells at a ratio of 1 : 2 (total of 9 × 10^6^ cells) and subjected to PEG fusion or PJI fusion as mentioned above. To assess DC–tumor cell vaccine functionality in tumor treatment challenge, 1 × 10^6^ viable B16‐F10 melanoma cells were injected intradermally on the backs of C57BL/6N mice. On days 2 and 7, tumor‐inoculated mice were given 2 doses of the DC–tumor hybrid cell vaccine intradermally on the opposite flank. Tumor growth was observed over time, and the tumor volume was measured in a blinded manner using slide calipers and was calculated using the following formula: tumor volume (mm^3^) = length × (width)^2^/2.

### ELISpot assay

Spleens from DC–tumor cell fusion vaccine‐treated mice were isolated 10 days after the last DC–tumor cell fusion vaccine injection. Splenocytes were isolated from the spleens as described previously. To stimulate splenocytes, B16‐F10 melanoma cells were treated with 15 μg·mL^−1^ mitomycin C (Nacalai Tesque Inc.) for 45 min. Splenocytes harvested from vaccine‐treated mice and mitomycin C‐treated B16‐F10 melanoma cells were mixed at a ratio of 10 : 1 and co‐cultured for 48 h. Nonadherent splenocytes were collected, and ELISpot assay was performed using the Mouse IFN‐γ Development Module (R&D Systems, Minneapolis, MN, USA) and the ELISpot Blue Color Module (R&D Systems). The numbers of IFN‐γ‐secreting cells were subsequently counted.

### Hybridoma production

C57BL/6N mice were injected with 10 μg OVA recombinant protein (Wako) mixed with alum adjuvant (Thermo Fisher Scientific) on days 0 and 14. On day 28, mouse splenocytes were harvested and fusion with NS‐1 myeloma cells was performed using the PEG or PJI method as mentioned above. After fusion, the cells were cultured in HAT selection medium (RPMI1640 HAT Supplement 1×) (Thermo Fisher Scientific) supplemented with 20% Hyclone fetal bovine serum (Cytiva, Washington, DC, USA), 0.1 mg·mL^−1^ penicillin–streptomycin mixed solution (Nacalai Tesque Inc.) and 10% Hybridoma Cloning Supplement (Santa Cruz Biotechnology, Santa Cruz, TX, USA) for 1 week. Hybridoma colonies were observed using the BZ‐X710 microscope (Keyence Corporation), and hybridoma cell fusion success rate was calculated by observing total number of wells with hybridoma presence.

### Statistical analysis

Statistical analyses were conducted using Student's two‐tailed unpaired *t*‐test with graphpad (Boston, MA, USA), and *P*‐values < 0.05 were considered statistically significant.

## Results

### Using instantaneous pressure to modify PEG fusion method

**Fig. 1:**
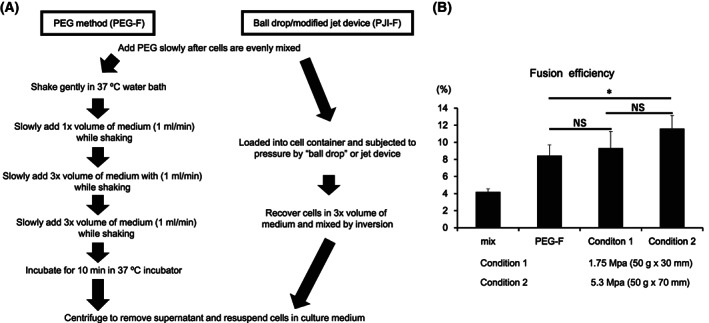
Standard PEG, modified PEG fusion process and cell fusion efficiency. (A) Fusion process by PEG fusion method (left) and the modified PEG fusion process by ‘ball drop’ method or jet injector method (right). (B) After PEG was added to NS‐1 myeloma and mouse splenocyte cell mixture, cell fusion was carried out either without shaking or incubation (Mix), by standard PEG fusion (PEG‐F) method or by ‘ball drop’ method at two pressure conditions, as labeled in figure. After cell fusion, all groups were analyzed by flow cytometry (*n* = 4 each). Data are expressed as the mean ± SD. *P*‐values were analyzed by a two‐tailed Student's *t*‐test. * indicates *P* < 0.05. NS indicates not significant.

The conventional PEG‐mediated cell fusion (PEG‐F) method is a commonly used method for cell fusion. Although it has the advantage of economical convenience, there is room for improvement with regard to the inconsistent and low fusion efficiency [25]. Previous research has shown that physical pressure can shorten the cell‐to‐cell distance [26], which promotes cell contact. So, we hypothesize whether application of acute pressure on cells can replace the cell shaking process in PEG‐F. To test this hypothesis, it was necessary to design a simple setup to generate acute pressure on cells. We designed a ‘ball drop’ apparatus (Fig. S1C). The rationale for this mechanism is when the ‘ball’ or weighted object is released from the top, the impact on the cell fusion container below would generate an instantaneous pressure on the cells inside the container. We also prepared two different ‘ball’ weights to generate different impact pressures to see how different pressures can affect cell fusion efficiency. In comparison with the standard PEG‐F method where PEG was first slowly added to NS‐1 myeloma cells and mouse splenocytes, then gently shaken for 20 min to promote cell‐to‐cell contact for cell fusion, in our new method, PEG‐added NS‐1 myeloma cells and mouse splenocyte mixture was directly loaded into and subjected to instantaneous pressure using our apparatus (Fig. 1A). Following cell fusion process, all cell mixtures were recovered and analyzed for fusion‐positive cells using flow cytometry. We found that acute pressure from falling weights was sufficient to induce cell fusion and is at least comparable with the standard PEG‐F method despite the shorter fusion time (Fig. 1B and Fig. S2). So, this suggests that pressure can promote cell fusion and potentially shorten the standard process.

### Jet injector as a pressure generator in PEG‐F modification

**Fig. 2:**
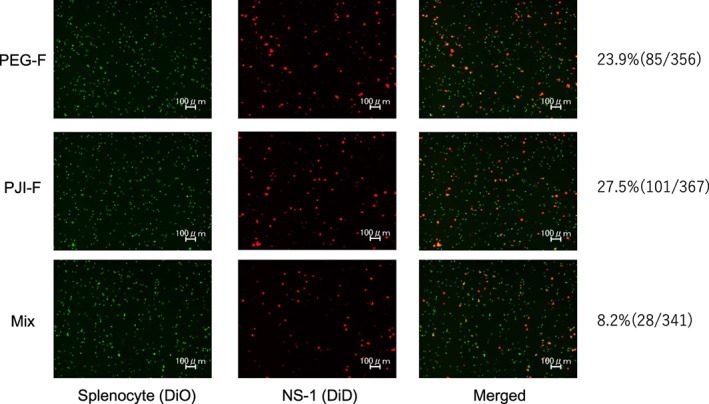
Splenocyte fusion with NS‐1 cell using jet device with PEG method. Cell fusion was performed using mouse splenocytes (DiO: green fluorescence) and NS‐1 cells (DiD: red fluorescence) with PEG either without shaking/incubation (Mix), by standard PEG‐F or by pyro‐drive jet injector‐mediated fusion (PJI‐F) method. After fusion, presence of fused cells was confirmed by fluorescence microscopy. Microscopy at 10× magnification.

Next, to further explore the use of pressure to aid in cell fusion, we designed a more refined apparatus setup capable of generating various pressure conditions to evaluate optimal conditions for cell fusion. Pyro‐drive jet injector (PJI) is a needleless injection device that uses controlled gunpowder ignition‐powered momentum to propel a plunger fitted to a syringe‐like container to instantaneously eject its preloaded contents through the skin. Injection pressure can be varied by adjusting gunpowder ratios in the device. We modified the PJI as a pressure generator source and replaced the container with one that is closed system to hold cells for cell fusion (Fig. S1A,B). To test whether the modified PJI apparatus can facilitate cell fusion, NS‐1 cells and mouse splenocytes were mixed with PEG and applied to the device. Fused cells were observed after PJI‐mediated fusion (PJI‐F), similar to PEG‐F method, showing that the modified PJI device can be used to complement PEG to induce cell fusion (Fig. 2 and Fig. S3).

### Positive correlation between pressure intensity and fusion efficiency

**Fig. 3:**
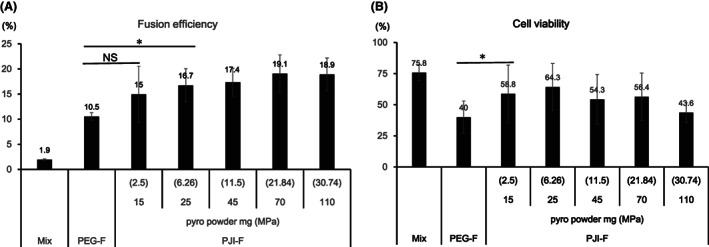
Increased pressure by PJI enhanced cell fusion efficiency, but excessive pressure reduced cell viability. (A) Cell fusion efficiency of fluorescent stained NS‐1 (DiR) and mouse splenocytes (CD45‐BV421) using standard PEG‐F method or PJI‐F method fitted with different doses of pyro powder (15–110 mg) corresponding to generating a range of different pressure conditions (2.5–30.7 MPa) and then analyzed by flow cytometry. (B) Cell viability in fused cell mixture after cell fusion described in (A) analyzed by trypan blue staining. Both cell fusion efficiency and cell viability results are graphed (*n* = 4 per group), and mean values are indicated above each column. Data are expressed as the mean ± SD. *P*‐values were analyzed by a two‐tailed Student's *t*‐test. * indicates *P* < 0.05. NS indicates not significant.

**Fig. 4:**
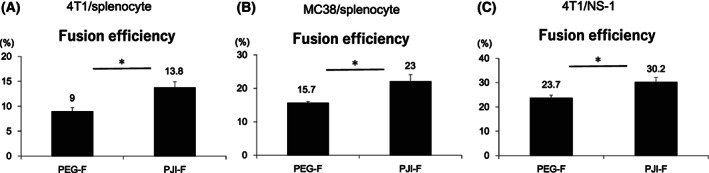
Cell fusion efficiency by PEG‐F and PJI‐F on different cell types. (A) Fusion of 4T1 cells and mouse splenocytes, (B) MC38 cells and mouse splenocytes, and (C) 4T1 cells and NS‐1 cells using standard PEG‐F method or PJI‐F (6.26 MPa) method; then, cell fusion efficiency was analyzed by flow cytometry. All cell fusion results are graphed (*n* = 4 per group), and mean values are indicated above each column. Data are expressed as the mean ± SD. *P*‐values were analyzed by a two‐tailed Student's *t*‐test. * indicates *P* < 0.05. NS indicates not significant.

To evaluate the effect of pressure on cell fusion efficiency, we prepared a range of PJIs with increasing amounts of pyro powder (15–110 mg), which produce corresponding pressure ranging from 2.5 to 30.7 MPa. PJI‐F method resulted in higher numbers of fused cells across all tested pressure conditions, in comparison with PEG‐F method (Fig. 3A). Cell viability after the fusion process is also crucial in the evaluation of cell fusion, due to potential cytotoxicity of PEG. We found that cell viability was higher using the PJI‐F method across all tested pressure conditions compared with the standard PEG‐F method, although PJI‐F cell viability is reduced when exposed to higher pressure conditions than compared to lower pressure conditions (Fig. 3B). Standard PEG‐F fusion efficiency is dependent on PEG concentration used; we also found that even at lower PEG concentration, PJI‐F was still more efficient than PEG‐F (Fig. S4). From these results, we have demonstrated that PJI‐F is both more efficient and likely less cytotoxic than standard PEG‐F method and that the acute pressure caused by PJI is sufficient to improve cell fusion efficiency. Additionally, we also applied PJI‐F to fuse several different combinations of cells aside from NS‐1/mouse splenocytes. We used PEG‐F and PJI‐F methods to fuse mouse mammary cell line 4T1 with mouse splenocytes, mouse colon cancer cell line MC38 with mouse splenocytes and 4T1 with NS‐1 (Fig. 4). We found that although fusion efficiency varied between different cell combinations, PJI‐F was still more efficient than PEG‐F in inducing cell fusion in all tested groups.

### PJI‐F can produce viable DC vaccine

**Fig. 5:**
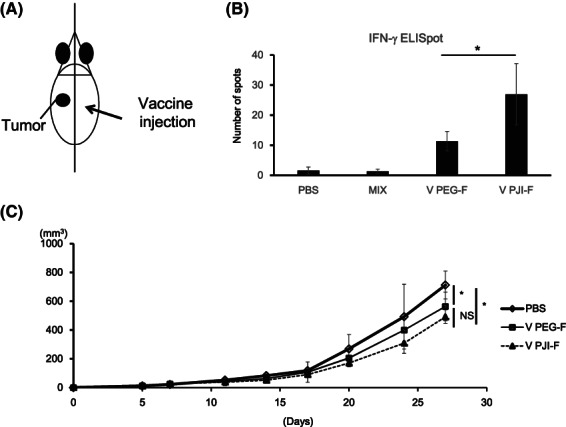
PJI‐F‐generated DC–tumor cell fusion vaccine elicited antitumor effect. (A) Schematic diagram of DC vaccine mouse tumor model challenge. C57BL/6N mice were subcutaneously injected on the left side with 2 × 10^5^ B16‐F10 melanoma cells. DC vaccines were intradermally administered on the opposite side on days 5 and 10. (B) DC–tumor cell fusion vaccine (DC vaccine) was generated by fusing mouse bone marrow‐derived splenocytes with B16‐F10 melanoma cells via nonshaken PEG method (Mix), PEG‐F method (V PEG‐F) or PJI‐F method (V PJI‐F). Naive C57BL/6N mice were immunized with DC vaccine and mouse splenocytes from each group (*n* = 4) were harvested 10 days later. Tumor‐specific IFN‐γ‐secreting T cell activation was analyzed using IFN‐γ ELISPOT assay. Nonvaccine‐treated mice (PBS) were used as a control group. * indicates *P* < 0.05. (C) Tumor size after DC vaccine administration was monitored (all *n* = 5). Tumor volumes are shown as mean ± SD. *P*‐values were analyzed by a two‐tailed Student's *t*‐test. * indicates *P* < 0.05. NS indicates not significant.

Besides fusion efficiency and cell viability, it is also important to assess the functionality of fused cells by PJI‐F method. Cell fusion is commonly used in the production of DC vaccines (DC–tumor cell) and antibody‐producing hybridomas (B cell‐myeloma cell). To properly evaluate PJI‐F method for cell fusion, successfully fused cells by PJI‐F method should function no different from those made using the standard PEG‐F method. In DC–tumor cell fusion vaccines (DC vaccines), DCs gain tumor‐associated antigens from fusing with tumor cells and process them for immune presentation to other immune cells, leading to immune activation against tumor cells. We produced DC vaccine by fusing DCs with B16‐F10 melanoma cells using the PJI‐F method. To test whether PJI‐F DC vaccine can stimulate the activation of tumor‐specific immune response, we injected DC vaccines into naive C57BL/6N mice (Fig. 5A) and then harvested splenocytes from these vaccinated mice 10 days later. The splenocytes were restimulated with inactivated B16‐F10 cells and analyzed using IFN‐γ ELISpot assay. Our results showed that PJI‐F DC vaccine induced significantly more tumor‐specific IFN‐γ‐secreting splenocytes in naive mice compared to those made by PEG‐F (Fig. 5B). This suggests that PJI‐F DC vaccine can stimulate potent tumor‐specific immune activation. Next, to see whether these DC vaccines can affect tumor growth, we first inoculated C57BL/6N mice with B16‐F10 tumor cells on their left flank. Five days after, we challenged DC vaccine on the right flank two times at a four‐day interval. We found that both PJI‐F and PEG‐F DC vaccines suppressed B16‐F10 tumor growth (Fig. 5C). From these results, we have demonstrated that PJI‐F can produce viable DC vaccine that has antigen presentation function and can elicit antitumor effect.

### PJI‐F method can produce hybridoma more efficiently than PEG‐F method

**Fig. 6:**
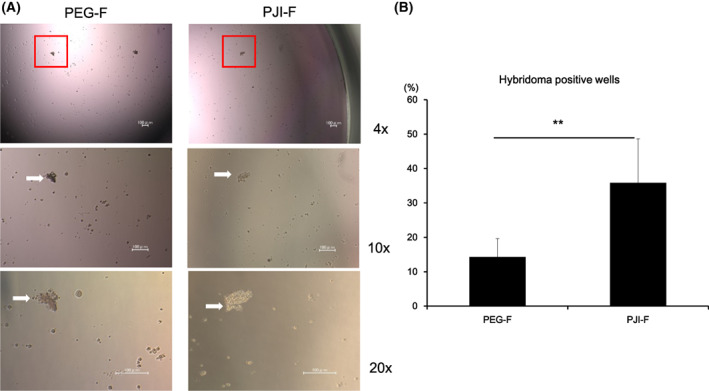
Hybridoma generation by PJI‐F method. (A) Hybridomas were generated by fusing NS‐1 myeloma cells with mouse splenocytes from recombinant OVA‐immunized BALB/c mice using standard PEG‐F method or PJI‐F method and cultured in HAT selection medium for 1 week. After HAT selection, hybridoma colony morphology was observed under the microscope. (B) Hybridoma generation efficiency was calculated by total number of hybridoma‐positive wells per plate, and results are represented graphically (*n* = 10). Data are expressed as the mean ± SD. *P*‐values were analyzed by a two‐tailed Student's *t*‐test. ** indicates *P* < 0.001.

Additionally, we also evaluated the use of PJI‐F method in the application of hybridoma production. To produce hybridoma, we fused NS‐1 myeloma cells and recombinant OVA peptide‐vaccinated mouse splenocytes using PJI‐F or PEG‐F method and then cultured the cells in HAT selection medium for 1 week. We observed that PJI‐F hybridoma colonies were indistinguishable from those from PEG‐F method (Fig. 6A) but PJI‐F method produced significantly more wells containing hybridoma colonies compared with PEG‐F method (> 2.5 fold) (Fig. 6B), indicating that PJI‐F method can produce hybridoma more efficiently than PEG‐F method. Therefore, PJI‐F performed better than PEG‐F in both fusion cell vaccine and hybridoma production. Based on all our findings, PJI‐F is a reliable method to improve cell fusion applications.

## Discussion

With the rapid development of antibody‐based drugs and regenerative medicine, cell fusion technology will become increasingly important and commonplace. PEG‐F may be an imperfect method, but the cost‐effectiveness is an attractive factor. One main problem of PEG‐F is the low cell fusion efficiency. Cell fusion efficiency using PEG‐F method is strongly dependent on cell type and skill proficiency [25]. In this research, we attempted to improve the efficiency of cell fusion using PEG method and reduce skill disparity by making the fusion process more standardized and consistent. To resolve these problems, we replaced the skill‐dependent operation (shaking process) during the PEG fusion process with the use of simplified steps (instantaneous pressure) to facilitate cell fusion (Fig. 1). Although effective cell fusion rates may vary between cell types, our results showed PJI‐F can achieve approximately 25–55% higher fusion efficiency than PEG‐F method (Figs 3A and 4). One potential reason for the variability of fusion efficiency in different types of cells may be related to cell size. For example, of the four types of cells used in our fusion experiments, we found that cell sizes are as follows: MC38 > NS‐1 > 4T1 > splenocyte using flow cytometry analysis (Fig. S5). These findings, along with the fusion efficiencies of specific combinations of these cells (Fig. 4), strongly suggest cell size may affect fusion efficiency. Based on our results, 4T1 fused with NS‐1, a larger sized cell, more efficiently than compared with fusion with smaller sized splenocytes.

The prolonged incubation with gentle shaking of cell mixture and PEG is the most critical step in the standard PEG cell fusion process. During this process, the mixed cells make essential cell‐to‐cell contact that promotes cell fusion, but the same conditions also affect overall cell viability due to PEG cytotoxicity. Therefore, to increase fusion cell viability, an important factor to consider would be reduction in the duration of cell contact with PEG. Since previous research indicated that high pressure causes molecular distance to shrink [27], we hypothesized whether application of instantaneous pressure could replace the shaking process during cell fusion (Fig. 1). Using jet device to generate pressure, we demonstrated successful cell‐to‐cell contact and detection of fused cells (Fig. 3 and Fig. S3). Although more detailed 3D examination of some ‘positive’ fused cells revealed false positives of close proximity cells rather than true fused cells (Fig. S3, white outlined arrows), other analytical approaches such as flow cytometry may be more conclusive in analyzing fusion efficiency (Figs 4 and 5). Cell fusion efficiency was positively correlated with increased pressure (Fig. 3A). However, excessively high pressure is detrimental for fusion process. Our results showed that when the pressurized output of the device is higher than 6 MPa, resultant cell viability started to decline. This indicates that stronger pressure may contribute to cell damage, so the balance between fusion efficiency and cell viability would determine optimal fusion conditions. Our results showed that the pressure of approximately 6 MPa is the best condition for cell fusion on our modified device. We also demonstrated that weak pressure (5.3 MPa) generated by ‘ball drop’ method was sufficient to boost cell fusion (Fig. 1B), but not as effective compared to low‐setting PJI (2.5 MPa) (Fig. 3A). One major difference between the ball drop method and PJI method, besides generated pressure, is the duration of pressurization. The free fall ball drop method exerted pressurization for a duration of approximately 0.8 ms, whereas PJI ignition exerted pressurization for < 0.5 ms. This suggests that pressurization duration may also affect overall fusion efficiency.

In standard PEG cell fusion method, the concentration of PEG used also affected cell fusion efficiency [17]. Our results showed that even when the amount of PEG‐1500 used was reduced to 25%, PJI‐F method improved cell fusion efficiency more effectively than PEG‐F method alone (Fig. S4). Therefore, instantaneous pressure can enhance cell fusion process regardless of PEG used. So, as long as optimal pressure conditions can be supplied, cell fusion using PEG can be improved, via similar pressure‐generating devices.

Here, we described using PJI‐F in two of the most common applications for cell fusion, DC vaccine and hybridoma production. Even though PJI‐F DC vaccine induced higher antigen‐specific immunity than PEG‐F DC vaccine, the overall functional antitumor effect was on par with PEG‐F DC vaccine in the tumor challenge model (Fig. 5B,C). This could be due to limitations of the fusion vaccine or the associated activated T cell effects alone in the complex tumor microenvironment. Both vaccines induced sufficient overall antitumor immune activation regardless of an increase in a specific subset of activated T cells arising in PJI‐F DC vaccine. But this overall significant suppression in tumor growth by both DC vaccines proves how effective DC vaccines could potentially be in antitumor therapy. And that application of pressure via PJI can improve the production of DC vaccines.

We have also shown that PJI‐F method could successfully be used to produce hybridomas. In hybridoma fusion, the resultant fused hybridoma cells should preserve cell properties of the parental cells, such as antibody production ability from immunized mouse splenocytes and immortal cell life span from the myeloma cell line. We found that PJI‐F method produced more than twice the number of hybridomas compared with standard PEG‐F method (Fig. 6A,B). This could be mainly due to the shorter cell fusion duration in the presence of PEG in the PJI‐F method, so cell viability is higher. Hence, PJI fusion method is a more efficient method for hybridoma production.

Recently, due to the emergence of COVID‐19, vaccines against this new infectious disease had been rapidly developed [28, 29, 30]. Taking advantage of the new vaccine research push, new types of vaccines, such as RNA or DNA‐based vaccines [31], have also come to the fore. Nucleic acid‐based vaccines can be developed rapidly, but often requires special carriers or injection methods to deliver these vaccines into cells [32, 33, 34, 35]. Jet injector is one of the many methods capable of injecting nucleic acid vaccines and has shown actual potential in delivering COVID‐19 vaccine in clinical settings [36, 37]. Besides being used as a delivery system, jet injector can also be used in other applications. Here, we have demonstrated a novel application of a modified jet device as a pressure generator to facilitate cell fusion. Although we did not compare the results of our PEG‐improved method with other existing fusion methods directly in this study due to disparate fusion conditions, we have shown that PJI‐F fusion method is an effective method over the traditional PEG fusion method.

In summary, instantaneous pressure could enhance cell fusion efficiency as well as simplify PEG fusion process and improve cell fusion applications such as DC fusion vaccines and hybridoma technology. Our findings showed that instantaneous pressure can complement PEG fusion and is a reliable method for existing and future applications involving cell fusion.

## Conflict of interest

The authors declare no conflict of interest.

## Author contributions

CYC was responsible for research project conceptualization, designed all experiments, collected data, and performed all analyses. JAT wrote the manuscript, involved in experimental discussion and troubleshooting, and obtained results for Figs 2A, 4 and 5. YS performed the double validation experiment and provided experimental assistance. TN involved in research discussions and preparation of microscopy samples. YH involved in ball drop experiment, equipment preparation and pressure measurement. KY involved in experimental assistance and animal management.

## Supporting information


**Fig. S1.** Cell fusion devices to generate pressure. (A) Closed system cell container with plunger to hold mixed cells with PEG for cell fusion. (B) Pyro‐drive jet injector device with cell container installed at one end of the instrument. (C) Schematic drawing of ‘Ball drop’ apparatus where a weighted ‘ball’ can be dropped and impact the plunger of the cell container docked at the bottom generating instantaneous pressure to cells within the container.
**Fig. S2.** Pressure‐generated fusion‐positive cells. Flow cytometry analysis of PEG‐mediated cell fusion of NS‐1 myeloma cells (DiR, APC‐A750) and mouse splenocytes (BV421, PB450) by ‘ball drop’ method as described in Fig. 1B. Double‐positive cells are fusion‐positive cells.
**Fig. S3.** Fused cell imaging. Detection of NS‐1 and mouse splenocyte fused cells by PJI‐F method using fluorescence microscope 3D imaging. (A) 20× magnification. (B) 40× magnification. (C) 40× magnification 3D image analysis. Successfully fused cells are indicated by solid white arrows, white outlined arrows show close proximity but fusion‐negative cells.
**Fig. S4.** PJI‐F method can enhance cell fusion even with reduction in PEG. Cell fusion efficiency when PEG was reduced to 25% in nonshaken PEG method (Mix), PEG‐F method and PJI‐F method (all *n* = 4). Data are expressed as the mean ± SD. *P*‐values were analyzed by a two‐tailed Student's t‐test. ** indicates *P* < 0.001. * indicates *P* < 0.05.
**Fig. S5.** Cell size differences affects cell fusion efficiency. Cell sizes of 4T1, MC38, NS‐1 and mouse splenocytes were analyzed by flow cytometry using FSC. Green line: splenocytes, orange line: NS‐1, blue line: 4 T1, and red line: MC38.

## Data Availability

The raw data supporting the conclusions of this study are available from the corresponding author upon reasonable request.
