# Supplementary material for: Enhancement of polyethylene glycol‐cell fusion efficiency by novel application of transient pressure using a jet injector
Source: FEBS Open Bio. 2023 Jan 27;13(3):478–89. doi: 10.1002/2211-5463.13557 (PMC9989930; doi:10.1002/2211-5463.13557)
Supplement: Supplementary file 1 — Fig. S1. Cell fusion devices to generate pressure. (A) Closed system cell container with plunger to hold mixed cells with PEG for cell fusion. (B) Pyro‐drive jet injector device with cell container installed at one end of the instrument. (C) Schematic drawing of ‘Ball drop’ apparatus where a weighted ‘ball’ can be dropped and impact the plunger of the cell container docked at the bottom generating instantaneous pressure to cells within the container. Fig. S2. Pressure‐generated fusion‐positive cells. Flow cytometry analysis of PEG‐mediated cell fusion of NS‐1 myeloma cells (DiR, APC‐A750) and mouse splenocytes (BV421, PB450) by ‘ball drop’ method as described in Fig. 1B. Double‐positive cells are fusion‐positive cells. Fig. S3. Fused cell imaging. Detection of NS‐1 and mouse splenocyte fused cells by PJI‐F method using fluorescence microscope 3D imaging. (A) 20× magnification. (B) 40× magnification. (C) 40× magnification 3D image analysis. Successfully fused cells are indicated by solid white arrows, white outlined arrows show close proximity but fusion‐negative cells. Fig. S4. PJI‐F method can enhance cell fusion even with reduction in PEG. Cell fusion efficiency when PEG was reduced to 25% in nonshaken PEG method (Mix), PEG‐F method and PJI‐F method (all n = 4). Data are expressed as the mean ± SD. P‐values were analyzed by a two‐tailed Student's t‐test. ** indicates P < 0.001. * indicates P < 0.05. Fig. S5. Cell size differences affects cell fusion efficiency. Cell sizes of 4T1, MC38, NS‐1 and mouse splenocytes were analyzed by flow cytometry using FSC. Green line: splenocytes, orange line: NS‐1, blue line: 4 T1, and red line: MC38. [file FEB4-13-478-s001.pdf]

# Enhancement of Polyethylene Glycol-cell fusion efficiency by novel application of transient pressure using Jet Injector

Supplementary figures

Supplementary Fig. 1

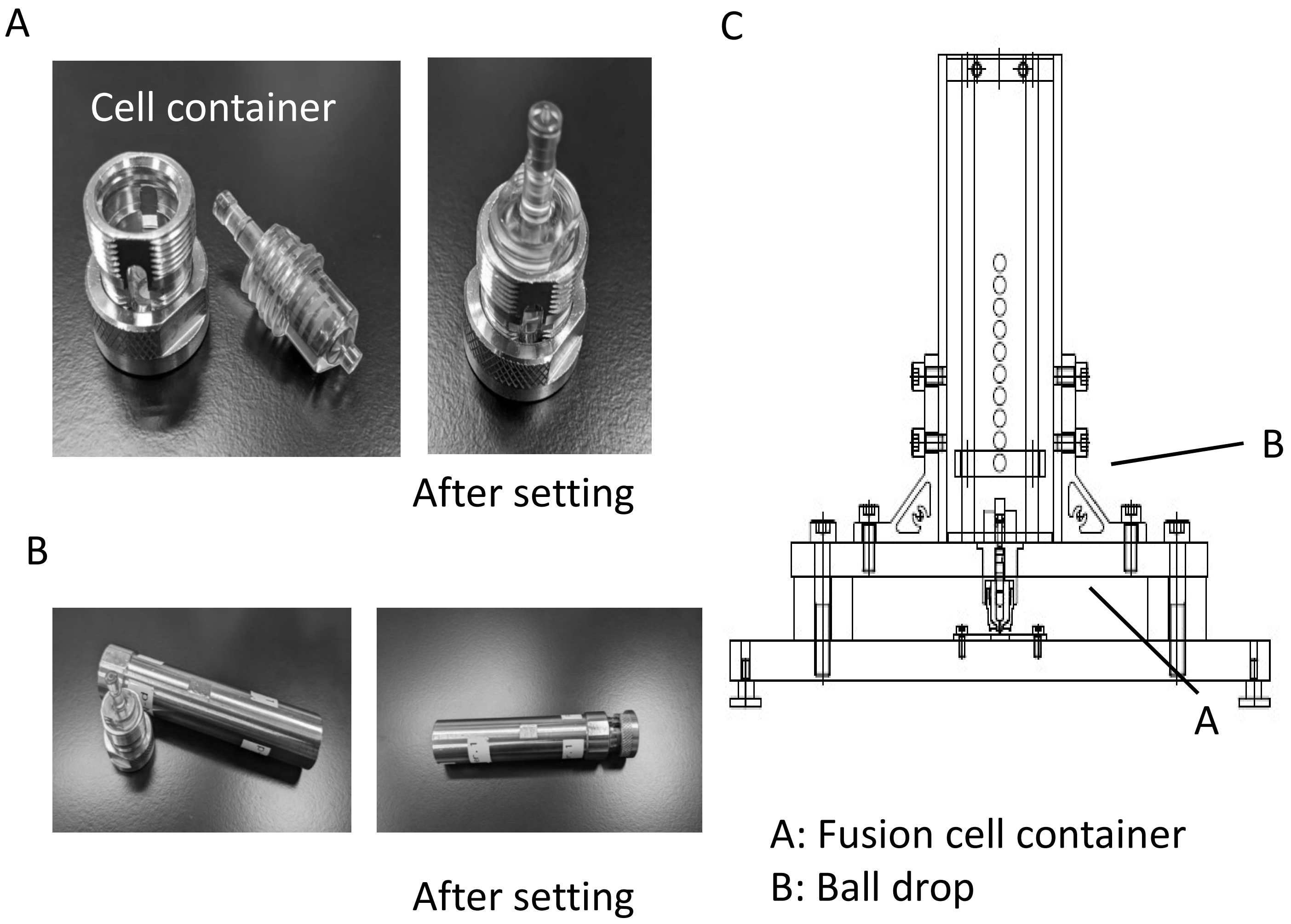


**Supplementary Figure 1. Cell fusion devices to generate pressure**
(A) Closed system cell container with plunger to hold mixed cells with PEG for cell fusion. (B) Pyro-drive jet injector device with cell container installed at one end of the instrument. (C) Schematic drawing of "Ball drop" apparatus where a weighted "ball" can be dropped and impact the plunger of the cell container docked at the bottom generating instantaneous pressure to cells within the container.

# Supplementary Fig. 2

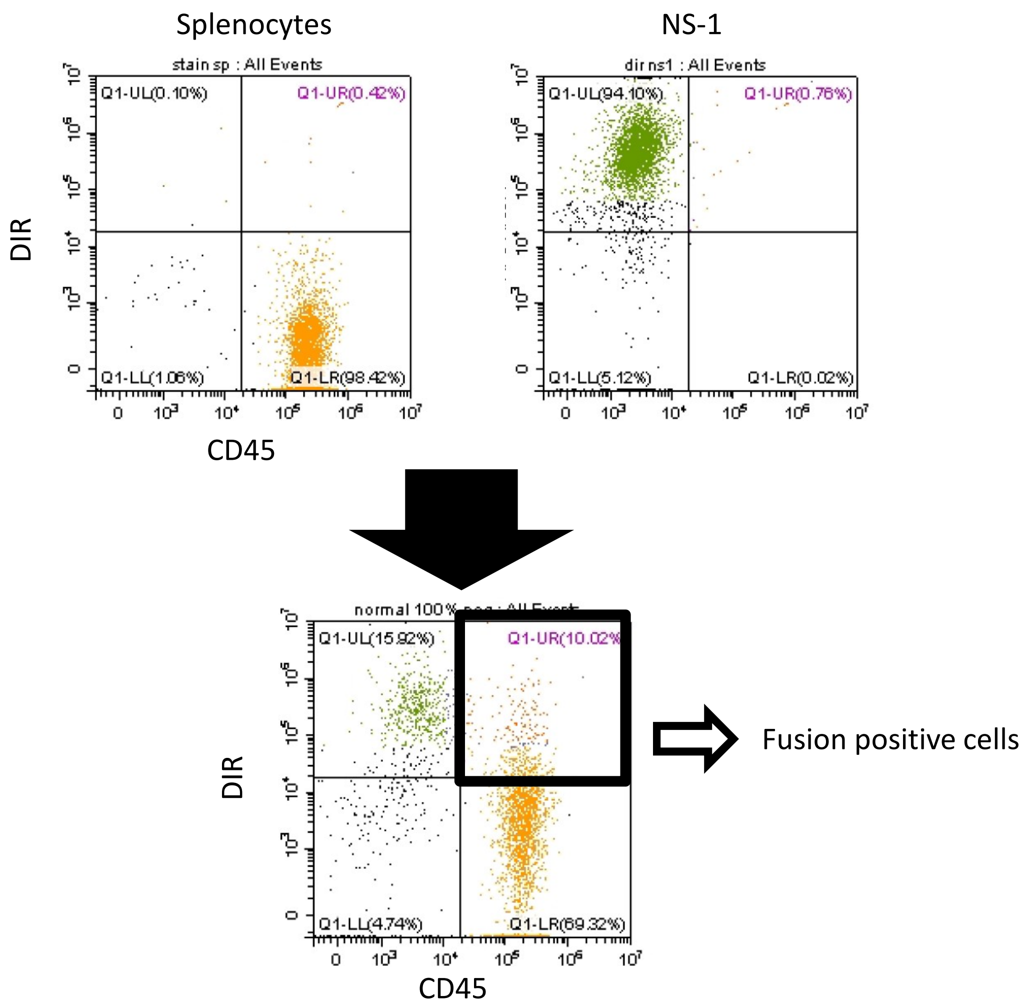


**Supplementary Figure 2. Pressure-generated fusion-positive cells**
Flow cytometry analysis of PEG-mediated cell fusion of NS-1 myeloma cells (DiR, APC-A750) and mouse splenocytes (BV421, PB450) by "ball drop" method as described in Fig. 1B. Double positive cells are fusion-positive cells.

Supplementary Fig. 3

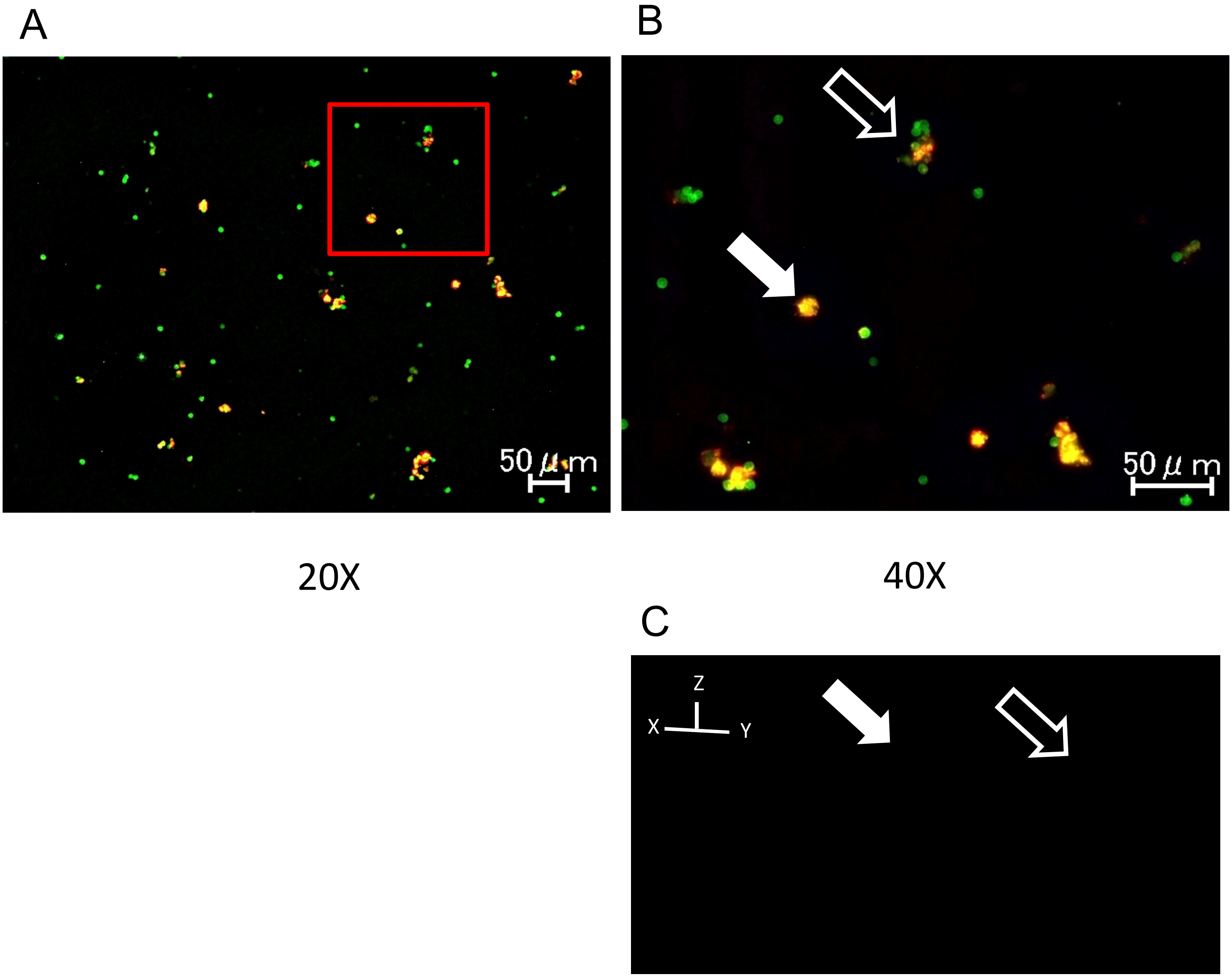


**Supplementary Figure 3. Fused cell imaging**

Detection of NS-1 and mouse splenocyte fused cells by PJI-F method using fluorescence microscope 3D imaging. (A) 20X magnification. (B) 40X magnification. (C) 40X magnification 3D image analysis. Successfully fused cells are indicated by solid white arrows, white outlined arrows show close proximity but fusion-negative cells. Scale bars: 50µm.

Supplementary Fig. 4

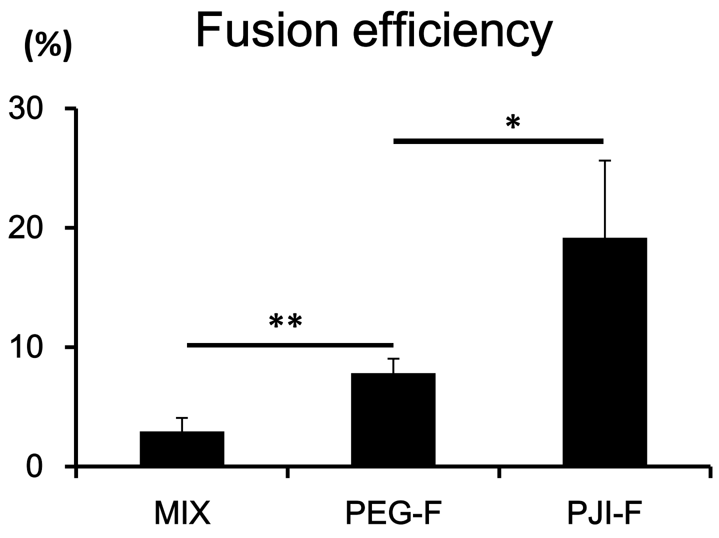


**Supplementary Figure 4. PJI-F method can enhance cell fusion even with reduction in PEG**
Cell fusion efficiency when PEG was reduced to 25% in non-shaken PEG method (Mix), PEG-F method and PJI-F method (all n = 4). Data are expressed as the mean ± SD. P values were analyzed by a two-tailed Student's t test. ** indicates $P<0.001$. * indicates $P<0.05$.

Supplementary Fig. 5

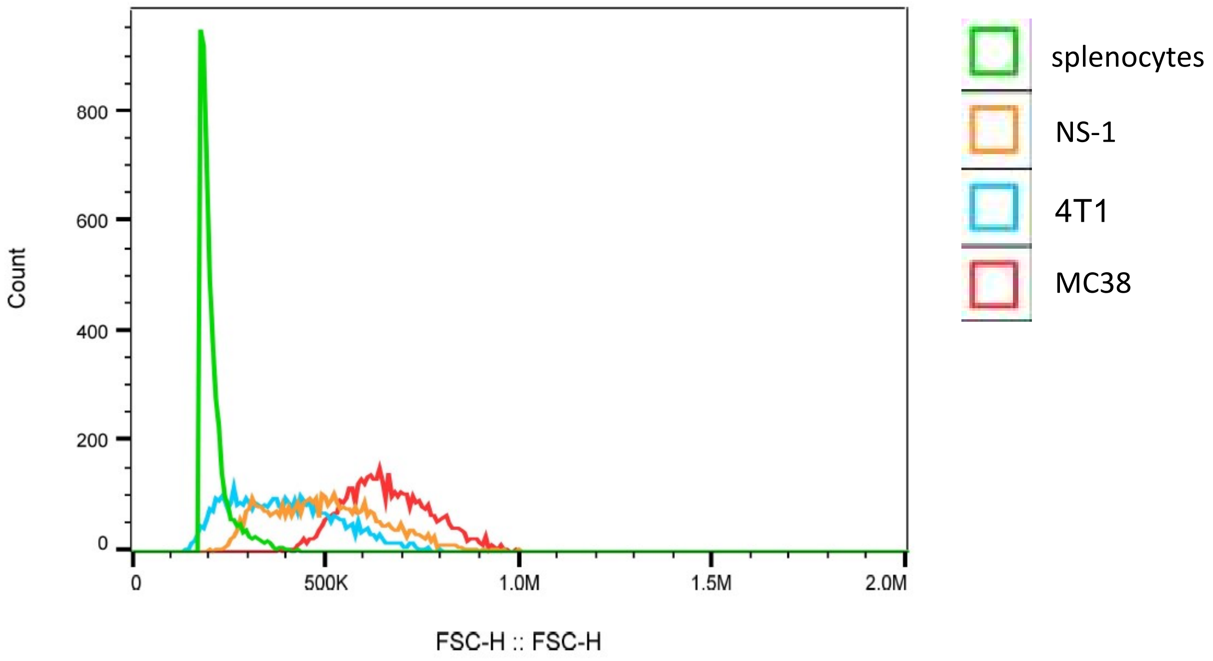


**Supplementary Figure 5. Cell size differences affects cell fusion efficiency**
Cell sizes of 4T1, MC38, NS-1 and mouse splenocytes was analyzed by flow cytometry using FSC. Green line: splenocytes, orange line: NS-1, blue line: 4T1 and red line: MC38.
